# Homing Receptor Expression Is Deviated on CD56^+^ Blood Lymphocytes during Pregnancy in Type 1 Diabetic Women

**DOI:** 10.1371/journal.pone.0119526

**Published:** 2015-03-20

**Authors:** Suzanne D. Burke, Alexandra V. C. Seaward, Heather Ramshaw, Graeme N. Smith, Sophia Virani, Barbara A. Croy, Patricia D. A. Lima

**Affiliations:** 1 Department of Biomedical and Molecular Sciences, Queen’s University, Kingston, Ontario, Canada; 2 Department of Obstetrics and Gynecology, Queen’s University, Kingston, Ontario, Canada; Medical Faculty, Otto-von-Guericke University Magdeburg, Medical Faculty, GERMANY

## Abstract

Type 1 Diabetes Mellitus (T1DM) is characterized by an augmented pro-inflammatory immune state. This contributes to the increased risk for gestational complications observed in T1DM mothers. In normal pregnancies, critical immunological changes occur, including the massive recruitment of lymphocytes, particularly CD56^bright^ NK cells, into early decidua basalis and a 2^nd^ trimester shift towards Type 2 immunity. Decidual CD56^bright^ NK cells arise at least partly from circulating progenitors expressing adhesion molecules SELL and ITGA4 and the chemokine receptors CXCR3 and CXCR4. *In vitro* studies show that T1DM reduces interactions between blood CD56^+^ NK cells and decidual endothelial cells by reducing SELL and ITGA4-based interactions. To address the mechanisms by which specific lymphocyte subsets may be recruited from the circulation during pregnancy and whether these mechanisms are altered in T1DM, flow cytometry was used to examine eight peripheral blood lymphocyte subsets (Type 1 (IL18R1^+^) and Type 2 (IL1RL1^+^) CD56^bright^ NK, CD56^dim^ NK, NKT and T cells) from control and T1DM women. Blood was collected serially over pregnancy and postpartum, and lymphocytes were compared for expression of homing receptors SELL, ITGA4, CXCR3, and CXCR4. The decline of Type 1/Type 2 immune cells in normal pregnancy was driven by an increase in Type 2 cells that did not occur in T1DM. CD56^bright^ NK cells from control women had the highest expression of all four receptors with greatest expression in 2^nd^ trimester. At this time, these receptors were expressed at very low levels by CD56^bright^ NK cells from TIDM patients. Type 1/Type 2 NKT cell ratios were not influenced by either pregnancy or TIDM. Our results suggest that T1DM alters immunological balances during pregnancy with its greatest impact on CD56^bright^ NK cells. This implicates CD56^bright^ NK cells in diabetic pregnancy complications.

## Introduction

Type 1 Diabetes Mellitus (T1DM) significantly elevates risk for gestational complications such as premature birth, macrosomia, perinatal death and preeclampsia [1–4]. These risks occur despite appropriate glycemic control [3]. Enhanced pro-inflammatory responses characterize not only T1DM [5,6], but also several pregnancy disorders [7–9]. During normal pregnancy, circulating and tissue-specific decidual lymphocytes contribute to conceptus tolerance, vascular remodeling and placental development [10,11]. In early human decidua (a transient, uterine stromal cell-derived progesterone-dependent tissue), the most abundant lymphocytes are CD56^bright^ Natural Killer cells (NK). Decidual CD56^bright^ (d)NK cells secrete cytokines and angiokines, but display limited cytotoxic ability [10,11]. Origins of CD56^bright^ dNK cells and other decidual lymphocytes are unclear. The possibility that they are derived from NK cell populations recruited from peripheral blood has been suggested [12–16]. However, research also supports recruitment at earlier lymphocyte stages or from other tissues as well as differentiation from resident self-renewing progenitors within the uterus [17]. Adoptive transfer studies in mice support all of these possibilities [18–20].

Previous research serially comparing cells from women at days 8 or 20 in a monitored menstrual cycle showed that CD56^+^ blood leukocytes collected at the LH surge have enhanced *in vitro* adhesion to endothelium of decidua basalis under shear forces [15]. Gains in *in vitro* adhesion to decidual endothelium are also reported in early pregnancy and have been linked with fertility [16]. In both studies, selectin L (SELL) and alpha 4 integrin (ITGA4)-based adhesion were identified as mechanisms promoting these changes in function [15, 16]. In contrast, blood CD56^+^ NK cells from pregnant T1DM and T2DM women are less adherent *in vitro* than blood CD56^+^ cells from gestational age-matched normal pregnant women to decidual endothelium, and more adherent to pancreatic endothelium [21]. SELL and ITGA4 alterations accounted for 75% of the functional change in diabetic CD56^+^ cell adhesion to decidual endothelium, suggesting that CD56^+^ NK cells in diabetic women had a diabetes-associated deviation in homing potential [21]. SELL and ITGA4 are strongly expressed by circulating CD56^bright^ NK cells [22] and have been identified as key receptors that could direct CD56^+^ blood NK cell homing to the uterus [22,23]. Leukocyte homing to specialized environments also involves interactions between tissue-secreted chemokines and leukocyte-expressed chemokine receptors [24–26]. Decidua produces abundant CXCL10 and CXCL12 [27], chemokines that bind to CXCR3 and CXCR4 respectively. Blood CD56^bright^ NK cells highly express CXCR3 and CXCR4 [24] that would enable lymphocyte-decidua interactions and promote lymphocyte retention within decidua. Stability in the expression of these receptors has not been assessed over pregnancy.

In the 1^st^ trimester of normal human pregnancy, a Type 1 immune state (also referred to as Th1 or the physiological pro-inflammatory state that normally characterizes men and non-pregnant women) is present systemically. In late 2^nd^ trimester, a shift towards Type 2 immunity (Th2 or anti-inflammatory state) occurs [28–30]. Here we compare the serial immune profiles (Type 1 or Type 2 immunity) and expression of selected homing receptors (ITGA4, SELL, CXCR3 and CXCR4) in blood lymphocyte subsets of normal pregnant women and T1DM women receiving regular prenatal medical care. Expression of IL18R1 or IL1RL1 was used to identify Type 1 and Type 2 lymphocytes, respectively, as previous described [30]. IL18R1 regulates IFNG [31], an important cytokine in early decidua [32]; as well as perforin expression in human decidual leukocytes, *in vitro* [33]. IL1RL1 through activation by IL33, a cytokine also expressed by early human decidua [34], is a crucial amplifier of innate rather than adaptive Type 2 immunity [34, 35].

We hypothesized that women with T1DM would have increased Type 1 lymphocyte bias throughout gestation and altered homing receptor expression on NK cell subsets. CD56^bright^ NK, CD56^dim^ NK, NKT and CD3^+^T cell subsets were examined in a cross-sectional longitudinal study that followed 7 T1DM patients and 8 age-matched controls over pregnancy and postpartum. Our outcome data show that in well-monitored T1DM women, the shift to Type 2 lymphocyte-predominance in the 2^nd^ trimester of pregnancy does not occur and the expression of homing receptors in blood lymphocytes is dampened compared with matched controls.

## Material and Methods

### Subject recruitment

Pregnant Caucasian women with T1DM (n = 7) and healthy, racially and age-matched controls (n = 8) were recruited at Kingston General Hospital (Kingston, ON, Canada). The T1DM women used insulin pumps and regularly visited the diabetic pregnancy clinic for supervised care. Exclusion criteria were multiple pregnancy, previous diagnosis or treatment of hypertension, polycystic ovarian syndrome, cancer and/or autoimmune disease other than T1DM. Patients provided a written consent and were free to withdraw at any time without any impact on their care. All patient samples were coded and blinded to investigators and no personal identifying information was recorded for study purposes. All protocols were pre-approved by the Queen’s University Health Sciences and Affiliated Teaching Hospitals Research Ethics Board. Blood samples were serially collected at 11–13 weeks (1^st^ trimester), 18–20 weeks (2^nd^ trimester) and 33–35 weeks (3^rd^ trimester) of gestation and after the 6^th^ postpartum week from each subject as available. These blood samples represent all of those used in our previous report on functional leukocyte adhesion *in vitro* [21] that contained sufficient cells for this second independent leukocyte assay.

### Sample collection

Blood was collected into acid citrate dextrose anti-coagulant by venipuncture (BDVacutainer, BD Bioscience; Mississauga, ON, Canada). Lymphocytes and plasma were immediately isolated by centrifugation (400 g; 30 min; 4°C) using Histopaque 1077 (Sigma Aldrich, St. Louis, MO, USA). Lymphocytes were collected, resuspended, washed thrice in phosphate buffered-saline (PBS), and then stained for flow cytometry.

### Flow cytometry

5 x 10^5^ cells/mL in 50μl of PBS containing 1% Bovine Serum Albumin (BSA) and 2% heat-inactivated human male serum were labeled using directly conjugated monoclonal antibodies and isotype controls. The antibodies and isotype controls, as well as the staining strategy are summarized in S1 Table and S2 Table respectively. After washing with 1% BSA in PBS and fixing with 2% paraformaldehyde, 1x10^5^ events were collected using a FC500 flow cytometer (Beckman Coulter, Mississauga, ON, Canada). Post-acquisition compensation and analysis were performed using FlowJo software (Tree Star, Inc., Ashland, OR, USA). Lymphocytes were analysed as CD56^bright^ NK, CD56^dim^ NK, NKT or T cells that expressed IL18R1 or IL1RL1 [30], followed by analysis of ITGA4, SELL, CXCR3 and CXCR4 expression.

### Multiplex cytokine/chemokines/growth factor microbead immunoassay

A multiplex biometric immunoassay, containing fluorescent microspheres conjugated with a monoclonal antibody specific for a target protein, was used for cytokine measurement according to the manufacturer's instructions (Bio-Plex Human Cytokine Assay # M50–0KCAF0Y; Bio-Rad Inc., Hercules, CA, USA). For this assay, six patients from the control group had plasma available. All seven plasma samples from T1DM patients were tested in this assay.

### Statistical analyses

Demographic data for subjects used in the flow cytometric analyses are presented as means ± SEM, unless otherwise specified, and compared by *t-test*. Lymphocyte subset data are presented as a percentage of total lymphocytes or percent positive of specified subset. Some data were log transformed to normalize their distribution and permit statistical analyses. Flow cytometry data were subjected to Grubb’s test to exclude outliers, followed by analysis between groups using two-way ANOVA with Bonferroni’s post-test. This was followed by one-way ANOVA with Bonferroni’s or Dunn’s post-tests, as applicable. To determine the impact of two T1DM patients who were diagnosed with preeclampsia after donation of their 3^rd^ trimester sample, analyses were repeated (as described above) excluding these patients from the T1DM group. The data from multiplex biometric immunoassay were tested for the normality using Shapiro-Wilk test, and compared using the Mann Whitney-U test.

Data were analyzed using Prism 5 Statistical Software package (GraphPad, San Diego, CA, USA). P values of <0.05 were considered statistically significant.

## Results and Discussion

### Patient demographics

Maternal demographics and clinical data are presented in Table 1. Two T1DM patients developed preeclampsia after donation of their 3^rd^ trimester sample. Both patients donated postpartum samples. The systolic blood pressure of T1DM patients was significantly higher than controls and the T1DM patients delivered preterm. Despite receiving regular pre-natal care at a high-risk, tertiary care hospital-based pregnancy clinic, glycemic control fluctuated. Using White’s classification of Diabetic Pregnancy, four T1DM were qualified as Class C and three were Class D (including both preeclamptic patients).

**Table 1:** Maternal and delivery demographics of control and T1DM patients.

	Control	T1DM	*P*
	(n = 8)	(n = 7)	
**Age (years)**	28.13 **±** 1.85	25.71 **±** 1.77	0.37
**Obstetrical History**			
Gravida	1.50 **±** 0.19	2.14 **±** 0.86	0.45
Term Deliveries	**0.50 ± 0.19**	**0.00 ± 0.00**	**0.03**
Preterm Deliveries	0.13 **±** 0.13	0.43 **±** 0.30	0.34
Pregnancy Loss	0.00 **±** 0.00	0.71 **±** 0.57	0.20
Living Children	0.38 **±** 0.18	0.14 **±** 0.14	0.35
**Delivery Information**			
GAD (weeks)	**39.80 ± 0.81**	**35.65 ± 1.48**	**0.05**
BW (g)	3749.11 **±** 230.82	3407.00 **±** 267.77	0.36
SVD	80%	57%	0.32
C-section	20%	43%	0.32
**Clinical Data**			
Systolic Blood Pressure (mmHg)	**115.13±2.06**	**132.26±2.97**	**0.02**
Diastolic Blood Pressure (mmHg)	73.16±1.47	72.74±2.32	0.87
Blood Glucose (mmol/L)	NAD	8.73 (**±** 1.16)	-
HbA1C (% of Hb)	NAD	7.54 (**±** 0.81)	-
Urine protein/24 hour (mg/dL)	3.08±2.30	21.47±17.49	0.39

Data are presented as mean ± SEM or percentage. GAD, gestation age at delivery. BW, birth weight of newborn. SVD, spontaneous vaginal delivery. C-section, Caesarean section, NAD, no abnormality detected. Normal ranges for blood glucose (3.3–5.6 mmol/L) and HbA1C (3.6–5.0% of Hb). Trace values for urine protein/24 hour are 5–20 mg/dL.

### Circulating lymphocytes across control and T1DM pregnancies

**Fig 1:**
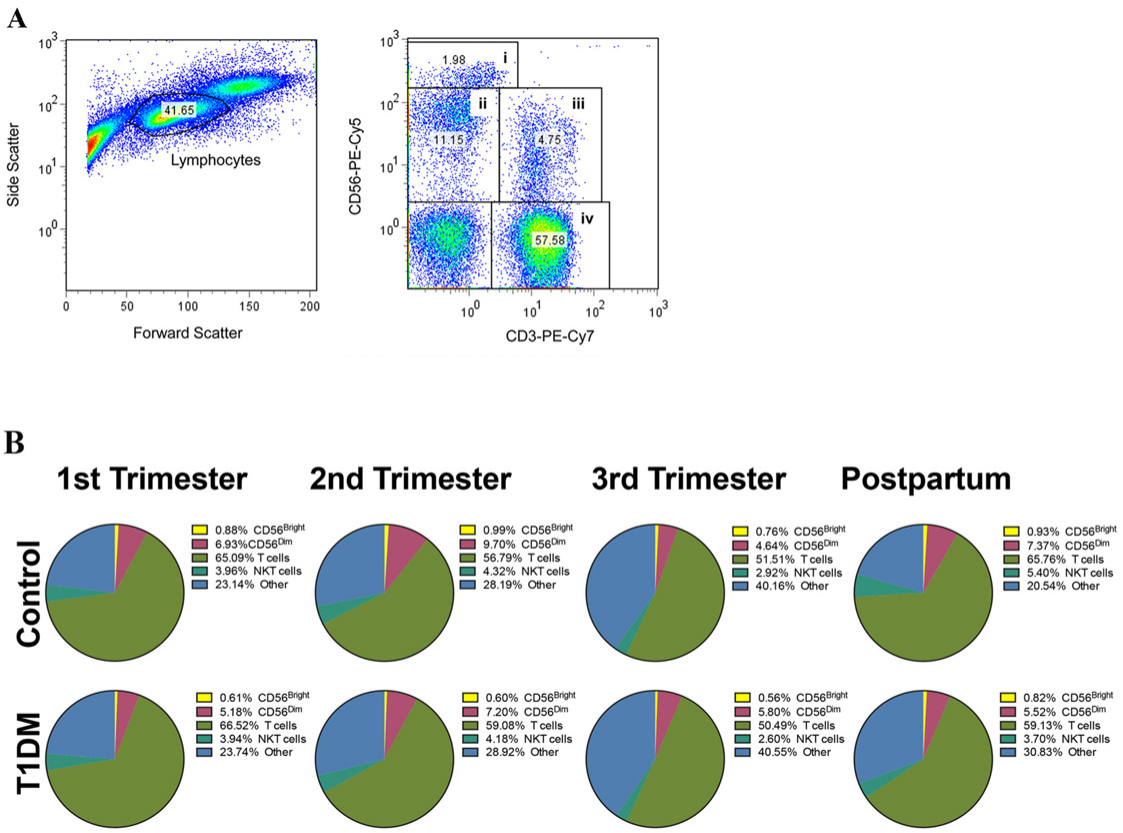
Quantification of blood lymphocytes by flow cytometry. (A) Total blood lymphocytes were gated by forward and side scatter (left histogram), and divided by CD3 and CD56 expression (right histogram): CD3^-^CD56^bright^ (CD56^bright^ NK cells), CD3^-^CD56^dim^ (CD56^dim^ NK cells), CD3^+^CD56^+^ (NKT cells) and CD3^+^CD56^-^ (T cells). (B) Pie-chart histograms represent the proportion of different lymphocyte subsets from total blood lymphocytes during pregnancy and postpartum in control (upper panel) and T1DM (bottom panel) patients. No significant changes were observed in the percentage of any lymphocyte subset over pregnancy and postpartum, or between groups (control *vs*. T1DM; P>0.05).

Lymphocytes were gated on forward scatter and side scatter properties. Based on CD3 and CD56 profiles, four lymphocyte subsets were analyzed: CD3^-^CD56^bright^ (CD56^bright^ NK cells), CD3^-^CD56^dim^ (CD56^dim^ NK cells), CD3^+^CD56^+^ (NKT cells) and CD3^+^CD56^-^ (T cells) (Fig. 1A). The proportions of NK CD56^bright^, NK CD56^dim^, NKT and T cells were invariant across pregnancy and postpartum and were not significantly different between control and T1DM patients (Fig. 1B).

### Type 1 *vs* Type 2 lymphocyte ratios across control and T1DM pregnancies

#### i) Global lymphocyte analyses

**Fig 2:**
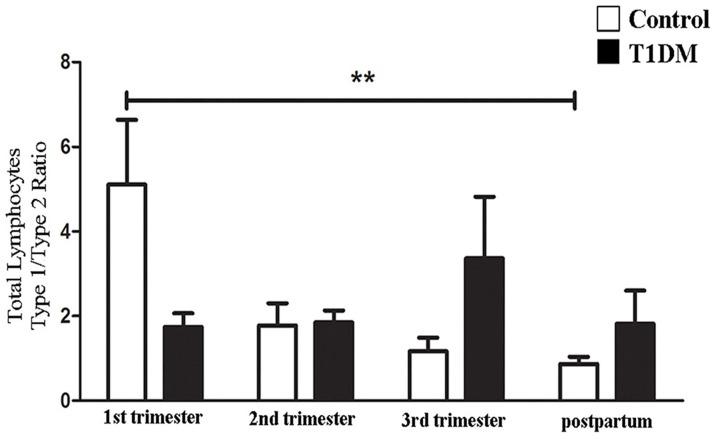
Type 1/Type 2 ratio (IL18R1+/IL1RL1+) of circulating lymphocytes. Type 1/Type2 cell ratio from total lymphocytes. In control patients, the linear trend post-test showed that the Type 1/Type2 cell ratio declined throughout pregnancy. Type 1/Type 2 cell ratios of T1DM patients did not differ across pregnancy or at postpartum (**P<0.01).

IL18R1 and IL1RL1 were used as surface molecules to identify Type 1 and Type 2 biased lymphocytes, respectively [30,31] and the ratio of Type 1/Type 2 lymphocytes was calculated. The Type 1/Type 2 lymphocyte ratios for control patients were highest in 1^st^ trimester and declined in later trimesters and postpartum (P = 0.0065; Fig. 2). The decline was driven by an increase in Type 2 lymphocytes. This shift of immunity from Type 1 (1^st^ trimester) towards Type 2 in later gestation is similar to data reported by others for normal human pregnancies [29,30]. In contrast, for T1DM patients, Type 1/Type 2 total lymphocyte ratios were invariant across pregnancy and postpartum (Fig. 2). Thus, no Type 2 immune shift occurred during pregnancy in T1DM patients.

#### ii) Lymphocyte subset analyses

**Fig 3:**
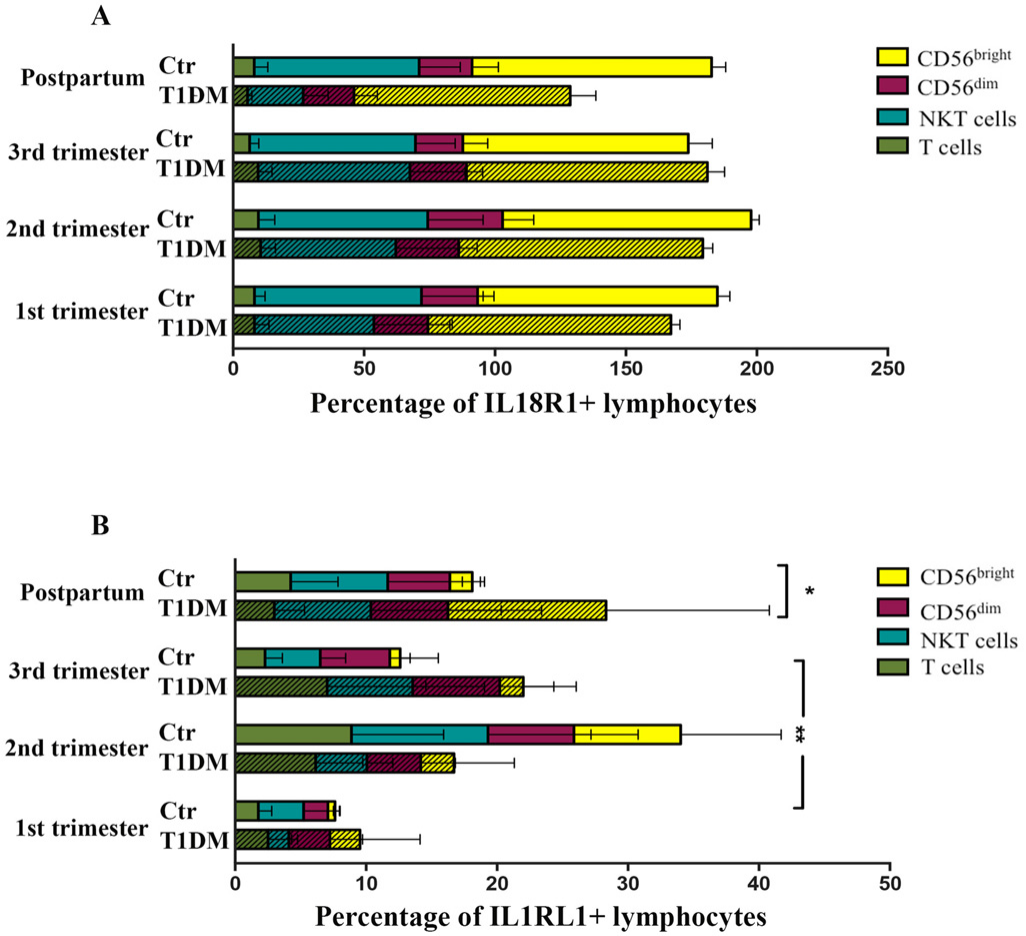
Type 1 lymphocyte (IL18R1^+^) and Type 2 lymphocyte (IL1RL1^+^) percentage. (A) The values are shown as percentage of Type 1 lymphocyte subsets in control (Ctr) and T1DM patients during pregnancy and postpartum. None of the Type 1 lymphocyte subsets (CD56^bright^ NK cells, CD56^dim^ NK cells, NKT cells and T cells) differ significantly across pregnancy or postpartum. (B) Percentage of Type 2 lymphocyte subsets (CD56^bright^ NK cells, CD56^dim^ NK cells, NKT cells and T cells) from control (Ctr) and T1DM patients during pregnancy and postpartum. Type 2 CD56^bright^ NK cells (*P<0.05), CD56^dim^ NK cells and T cells were significantly augmented in the 2^nd^ trimester compared to 1^st^ trimester from control, but not from T1DM patients. Differences also were detected in Type 2 CD56^bright^ NK cells between 2^nd^ and 3^rd^ trimester from control patients (*P<0.05); Comparison between control and T1DM patients demonstrated that at postpartum, differences were observed in the Type 2 CD56^bright^ NK cells (*P<0.05).

The proportions of lymphocyte subsets (CD56^bright^ NK, CD56^dim^ NK, NKT and T cells) expressing IL18R1 (Type 1 cells) did not differ significantly between control and T1DM patients over pregnancy and postpartum (Fig. 3A; S3 Table). However, Type 2 (IL1RL1^+^) CD56^bright^ NK, CD56^dim^ NK and T cells increased significantly in control patients in 2^nd^ trimester (Fig. 3B; S3 Table; P<0.05), while lymphocytes from T1DM patients expressing IL1RL1 were invariant over pregnancy and postpartum (Fig. 3B; S3 Table). NKT cells were invariant across pregnancy and postpartum in control and T1DM patients. These data suggest that specific subsets of CD56^bright^ NK, CD56^dim^ NK and T cells participate in the shift of immunity from Type 1 towards Type 2 in 2^nd^ trimester of normal pregnancy. They further indicate that by 18–20 weeks gestation in T1DM patients, a physiological change fails to occur amongst CD56^bright^ NK, CD56^dim^ NK and T cells.

**Fig 4:**
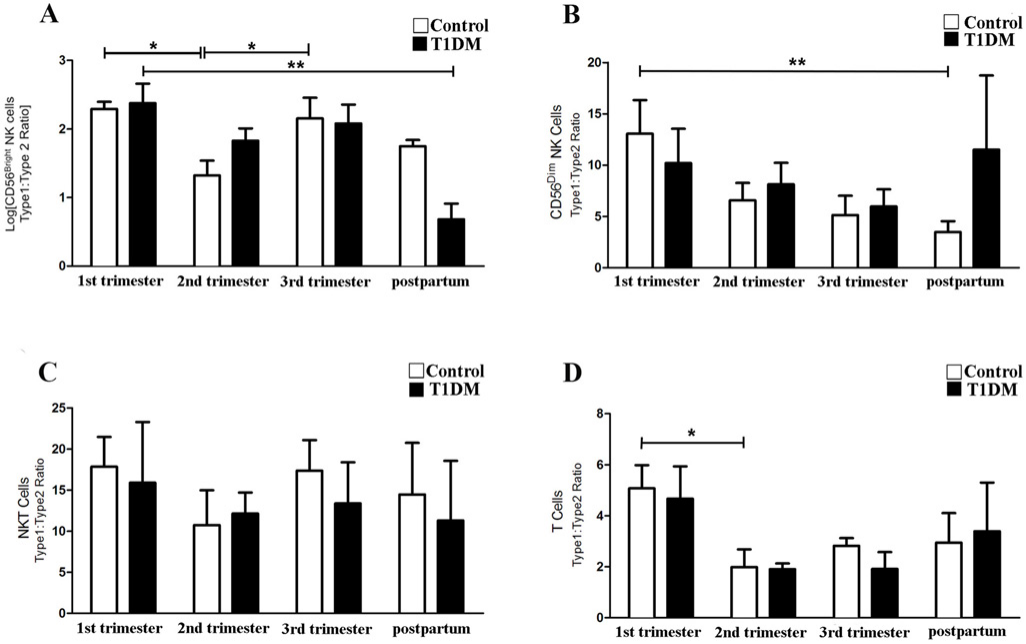
Type 1/Type 2 ratio (IL18R1^+^/IL1RL1^+^) of specific lymphocyte subsets. Type 1/Type 2 ratio of CD56^bright^ NK cells (A), CD56^dim^ NK cells (B), NKT cells (C) and T cells (D). The data are expressed as percentage following lognormal transformation (A) and percentage (B-D) of cells expressing IL18R1 /IL1RL1. (A) Type 1/Type 2 ratio of CD56^bright^ NK cells was significantly lower in 2^nd^ trimester compared with 1^st^ and 3^rd^ trimesters in control pregnancies (*P<0.05), but not in T1DM patients. (B) CD56^dim^ NK cells had significantly higher Type 1/Type 2 ratios in 1^st^ trimester control than postpartum samples (*P<0.05). CD56^dim^ NK cells Type 1/Type 2 ratio from T1DM patients did not significantly differ across pregnancy and postpartum. (C) Type 1/Type 2 ratios of NKT cells did not differ significantly across pregnancy and postpartum in control and T1DM patients, or between control and T1DM patients. (D) Type 1/Type 2 ratio for T cells was higher in 1^st^ than 2^nd^ trimesters in control pregnancies (*P<0.05), but not in T1DM patients.

Type 1/Type 2 ratios were also calculated for each lymphocyte subset as described below (Fig. 4A-D).

**CD56** ^**bright**^ **NK cells:** In control patients, the Type 1/Type 2 ratio of CD56^bright^ NK cell was lower in 2^nd^ compared to 1^st^ trimester, demonstrating the expected shift towards a Type 2 profile (P<0.05). At 3^rd^ trimester, the ratio was significantly higher than in 2^nd^ trimester (P<0.05). In T1DM patients, the Type 1/Type 2 ratio of CD56^bright^ NK cells was significantly higher in 1^st^ trimester than postpartum (P<0.05), but no differences were found comparing 1^st^, 2^nd^ and 3^rd^ trimesters. No significant differences in the Type 1/Type 2 ratio of CD56^bright^ NK cells were found between control and T1DM patients in any pregnancy or postpartum period (Fig. 4A).

**CD56** ^**dim**^ **NK cells**: In control patients, the Type 1/Type 2 ratio of CD56^dim^ NK cells declined from 1^st^ trimester to the postpartum period (P<0.05; Fig. 4B). In T1DM patients, no alterations occurred. No significant differences were found between control and T1DM patients for any pregnancy or postpartum period (Fig. 4B).

**CD3** ^**+**^ **CD56** ^**+**^ **NKT cells**: Type 1/Type 2 ratios did not differ across pregnancy and postpartum in either patient group or between patient groups (Fig. 4C). Thus, the Type 1/Type 2 NKT cell ratio in blood is not influenced by either pregnancy or T1DM.

**CD3** ^**+**^ **CD56** ^**-**^ **T cells:** In control patients, the Type 1/Type 2 ratio expressed by T cells was lower in 2^nd^ compared to 1^st^ trimester (P<0.05), consistent with a shift towards a Type 2 immune profile. A similar reduction was not observed in T1DM patients (Fig. 4D). No differences were observed when Type 1/Type 2 T cell ratios were compared between control and T1DM patients.

Exclusion of data from the two T1DM patients who subsequently became preeclamptic did not alter outcomes from these analyses. That is, Type 1/Type 2 ratios were stable for all lymphocyte subsets of the T1DM women. This suggests that T1DM, whether or not it is accompanied by preeclampsia, interferes with the normal gestational shift from Type 1 to Type 2 immunity in CD56^bright^ NK cells, CD56^dim^ NK cells and in T cells. This finding is also consistent with previous studies in preeclamptic, non-diabetic patients who demonstrated failure to convert from Type 1 to Type 2 cytokine bias [30]. This could represent a common immune pathway, or potential mechanism promoting diabetogenic risk for preeclampsia, but requires further study.

### Expression of adhesion and chemokine receptors across control and T1DM pregnancies and postpartum

The percentages of each lymphocyte subset expressing ITGA4 and SELL, and CXCR3 and CXCR4 were examined during pregnancy and postpartum. These data are summarized in S4 Table and S5 Table. CD56^bright^ NK cells were the subset with highest expression of each of the receptors and with the largest changes in homing receptor expression across pregnancy and postpartum.

**Fig 5:**
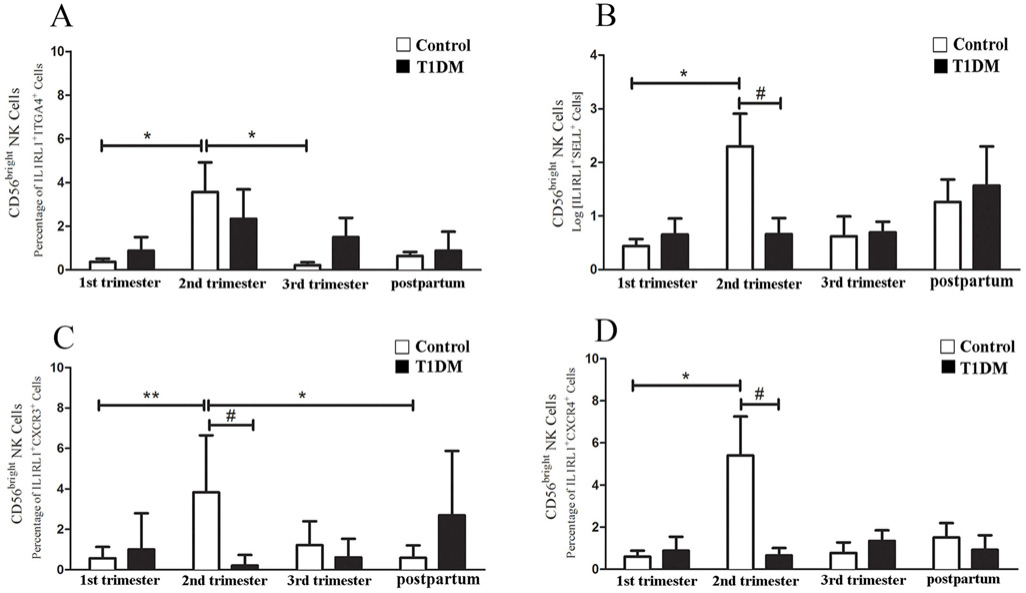
Homing receptor expression by Type 2 CD56^bright^ NK cells. In (A) the percentage of Type 2 CD56^bright^ NK cells expressing ITGA4 in control patients was significantly increased in the 2^nd^ trimester versus 1^st^ and 3^rd^ trimesters (*P<0.05). No significant differences were detected in T1DM patients. In (B) Log of and (C-D) percentage of Type 2 CD56^bright^ NK cells expressing SELL (B), CXCR3 (C) and CXCR4 (D) are shown. A significant increase in Type 2 CD56^bright^ NK cells expressing SELL (B), CXCR3 (C) and CXCR4 (D) was detected in 2^nd^ compared to 1^st^ trimester (*P<0.05; **P<0.01). Type 2 CD56^bright^ NK cells expressing CXCR3 were also significantly higher in 2^nd^ trimester than postpartum (*P<0.05). Control patients had more Type 2 CD56^bright^ NK cells expressing SELL (B), CXCR3 (C) and CXCR4 (D) than T1DM patients in 2^nd^ trimester (#P<0.05).

Type 2 CD56^bright^ NK cells were the lymphocytes showing the greatest variation in homing receptor expression in control pregnancies, suggesting that this subset has the greatest ability to home to tissues from blood. Type 2 CD56^bright^ NK cells from control women expressed ITGA4 at higher levels in 2^nd^ trimester than in 1^st^ or 3^rd^ trimesters (Fig. 5A). Type 2 CD56^bright^ NK cells from control patients also had greater expression of SELL, CXCR3 and CXCR4 during 2^nd^ trimester compared to 1^st^ trimester (Fig. 5B-D). In T1DM patients, few Type 2 CD56^bright^ NK cells expressed SELL, CXCR3 or CXCR4 at 2^nd^ trimester (Fig. 5B-D).

ITGA4 and SELL expression by circulating CD56^bright^ NK cells is widely known to be important for NK cell adhesion, interaction with endothelium and extravasation [15, 36, 37]. Our data suggest that extravasation of Type 2 CD56^bright^ NK cells is preferentially increased only during the 2^nd^ trimester of normal pregnancy while the potential for extravasation of Type 1 CD56^bright^ NK cells is high in both 1^st^ and 2^nd^ trimesters. In normal pregnancy, reduced expression of ITGA4 and SELL occurs in Type 1 and Type 2 blood NK cells during 3^rd^ trimester, an interval during which fewer CD56^bright^ NK cells are present in decidua [38]. The homing of CD56^bright^ NK cells into decidual tissue also involves CXCR3 and CXCR4 [12, 14, 27, 39]. T1DM-associated reductions in SELL and ITGA4 expression by Type 2 CD56^bright^ NK cells during 2^nd^ trimester may explain the observation that CD56^+^ blood lymphocytes from these same 2^nd^ trimester T1DM pregnancies had reduced *in vitro* binding to mouse decidual endothelium [21].

**Fig 6:**
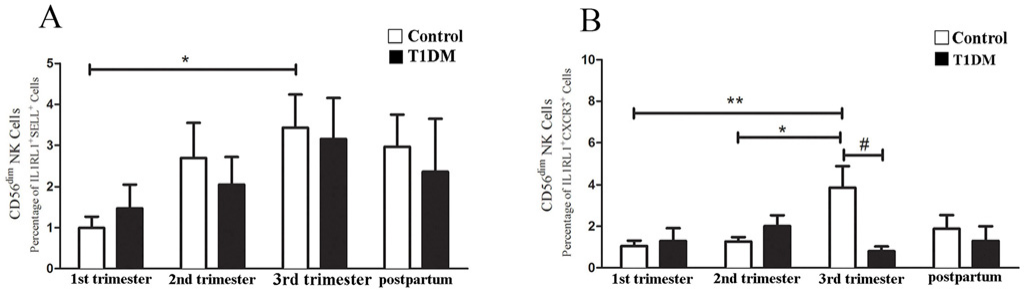
Homing receptor expression by Type 2 CD56^dim^ NK cells. Percentage of Type 2 CD56^dim^ NK cells expressing SELL (A) and CXCR3 (B). (A) Type 2 CD56^dim^ NK cells expressing SELL from control patients in 3^rd^ trimester were higher than in 1^st^ trimester (*P<0.05). No differences in Type 2 CD56^dim^ NK cells were detected over pregnancy in T1DM patients. (B) Control 3^rd^ trimester patients had a higher percentage of Type 2 CD56^dim^ NK cells expressing CXC3 than in 1^st^ (**P<0.01) or 2^nd^ trimesters (*P<0.05). The Type 2 CD56^dim^ NK cells from T1DM patients did not differ across pregnancy. Type 2 CD56^dim^ NK cells expressing CXCR3 in the 3^rd^ trimester were numerous in control than T1DM patients (#P<0.05).

Changes in homing receptor expression also occurred in Type 2 CD56^dim^ NK cells. In control patients, CD56^dim^ NK cells expressing SELL and CXCR3 were significantly higher in 3^rd^ than in 1^st^ or 2^nd^ trimesters (Fig. 6A, B), while in T1DM patients no changes were detected. Type 2 CD56^dim^ NK cells expressing CXCR3 were significant lower in T1DM than control patients at 3^rd^ trimester (Fig. 6B).

**Fig 7:**
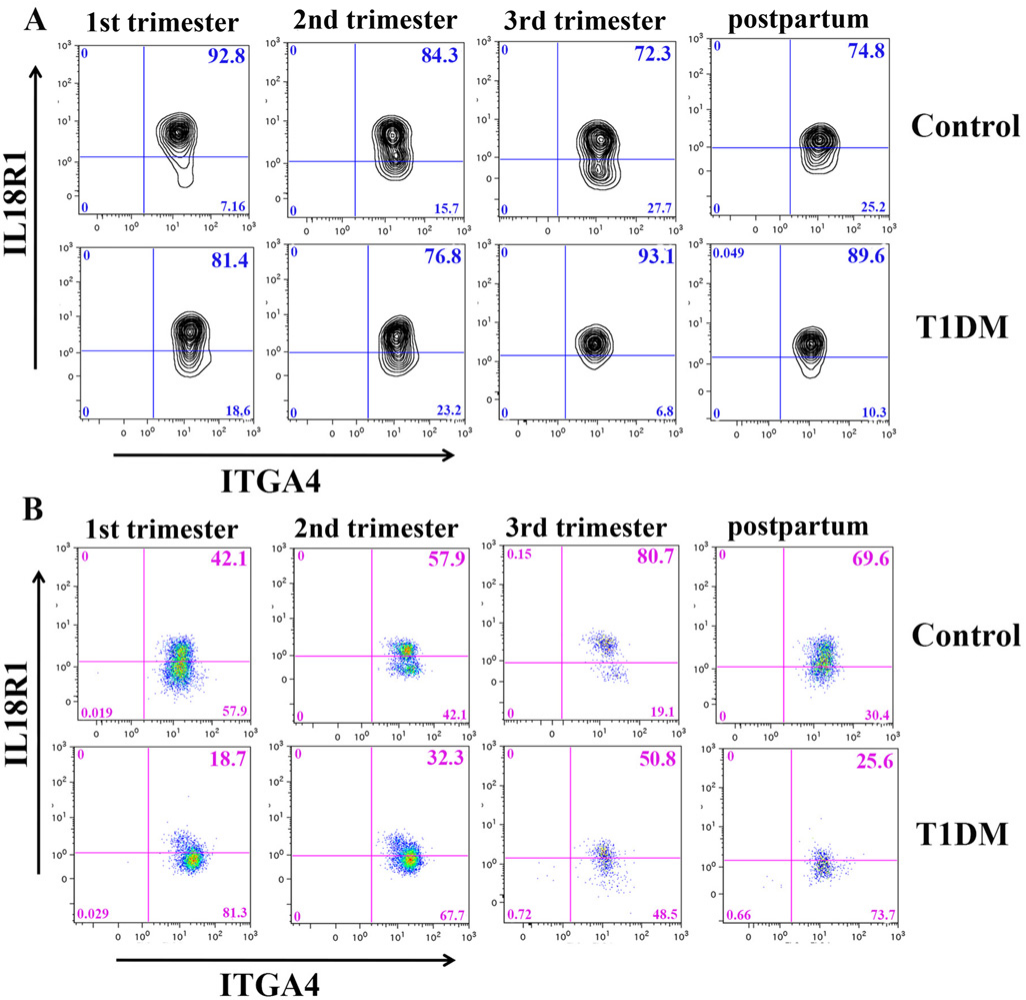
Homing receptor expression by Type 1 CD56^bright^ NK and NKT cells. (A) Representative contour plots of Type 1 CD56^bright^ NK cells expressing ITGA4 analysis from a control (upper panel) and a T1DM (bottom panel) patient are shown. (B) Representative pseudocolor dot plot of Type 1 NKT cells (CD3^+^CD56^+^) expressing ITGA4 from a control (upper panel) and T1DM (bottom panel) patient.

Two subsets of Type 1 lymphocytes varied in homing receptor expression: CD56^bright^ NK cells and NKT cells. Type 1 CD56^bright^ NK cells expressing ITGA4 were significantly higher in 1^st^ (92.4%±5.3) and 2^nd^ (94.8%±3.7) trimester than in 3^rd^ (78.4%±8.7) trimester from control pregnancies (P<0.05; Fig. 7A). Conversely, no alterations were observed in CD56^bright^ NK cell expression of any homing receptor across pregnancy and postpartum in T1DM patients. Comparisons between control and T1DM patients demonstrated that Type 1 CD56^bright^ NK cells expressing ITGA4 were more frequent in T1DM than in control patients in 3^rd^ trimester (78.4%±8.7 vs. 91.0%±5.5, P<0.05; Fig. 7A). This finding may suggest that in T1DM, the need for high levels of uNK cell functions persists at the maternal fetal interface longer (into 3^rd^ trimester) than in normal pregnancies and that a compensatory response has occurred to sustain CD56^bright^ NK cell extravasation. The timing of this difference also coincides with onset rapid fetal growth and of clinical recognition of preeclampsia and may reflect a lymphocyte phenotype induced by tissue-specific endothelial cell stress [30, 40–42].

The analysis of homing receptors expressed by Type 1 NKT cells showed that T1DM patients, but not control patients, vary during pregnancy. In T1DM patients, Type 1 NKT cells expressing ITGA4 were significantly reduced at postpartum (23.2%±11.4) compared to 3^rd^ trimester (63.5%±13.0; P<0.05, Fig. 7B). Comparisons between control and T1DM patients demonstrated that at 1^st^ trimester (69.1%±15.2 vs. 32.6%±19.9) and postpartum (56.5%±19.3 *vs*. 23.2%±11.4), fewer Type 1 NKT cells expressing ITGA4 were measured in T1DM than control patients (control vs. T1DM respectively, P<0.05; Fig. 7B). Exclusion of the two preeclamptic patients from these analyses annulled the statistical differences. ITGA4 involvement in NKT cell homing potential for decidua is compatible with its higher expression in peripheral NKT cells of fertile versus infertile women [43]. Since no control patients developed preeclampsia, the implications of the altered expression of ITGA4 on Type 1 NKT cells in preeclampsia remains unclear.

Adoptive transfer experiments in mice [18] and analyses of human decidua [44] supports the conclusion that decidual lymphocytes arise from cells that home from the circulation. Further, *in vitro* differentiation of CD56^+^ blood NK cells into decidua-like CD16^-^CD56^bright^ is reported [45]. However, endometrial hematopoietic progenitor cells (CD34^+^CD45^+^) are also described in first trimester [17]. Thus, at least in early decidua, hematopoietic progenitor cells may contribute significantly to the gains in decidual NK cells, as predicted by their capacity for *in vitro* differentiation [17]. Few published reports have addressed the presence or life span of decidual hematopoietic progenitor cells that can differentiate into decidual CD56^bright^ NK cells beyond 1^st^ trimester. Such studies are limited largely by ethical constraints. Bulmer and Lash [38] found high numbers of CD56^+^ uNK cells in placental bed biopsies until week 20 of pregnancy (2^nd^ trimester). The life span of uNK cells can only be surmised from estimates of peripheral NK cells. The half-life of human NK cells has been estimated at about 12 days [46]. This is similar to the estimated half-life of peripheral mouse NK cells [47,48]. The main differences between our normal and T1DM study participants were found in 2^nd^ trimester, a time that may represent a secondary wave of leukocyte recruitment to the decidua from the circulation. The enrichment of different lymphocyte subsets (functionally and phenotypically) in decidua as pregnancy progresses is widely accepted. The data presented here add to understanding that over the course of normal pregnancy, the potential for tissue recruitment of circulating NK and T cells is dynamic and differs between lymphocyte subsets, with CD56^bright^ NK cells showing the highest and most dynamic expression of key receptors. We also show that a disease process, T1DM, depresses these receptor changes in a manner that might reduce important lymphocyte-based functions at the maternal-fetal interface.

### Cytokines, chemokines and growth factors across control and T1DM pregnancies and postpartum

**Fig 8:**
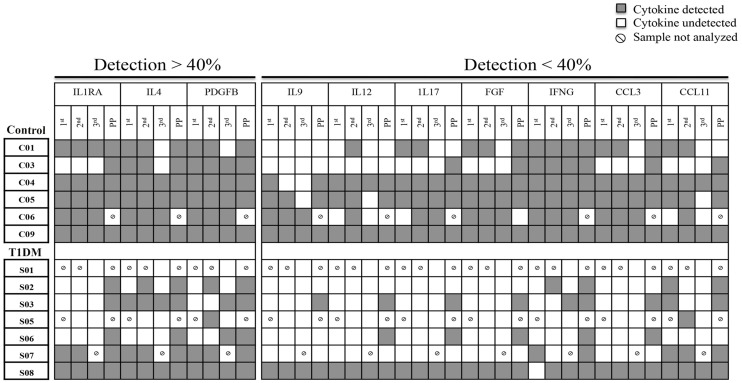
Detection of cytokines, chemokines and growth factors in control and T1DM patients across pregnancy and postpartum (PP). The representative scheme demonstrates 10 cytokines that were sporadically detected in the plasma from 13 patients: controls (C0) and T1DM (S0). Three cytokines were detected in a frequency higher than 40% (IL1RA, IL4 and PGFFB) while seven cytokines were detected in a frequency lower than 40% (IL9, IL12, IL17, FGF, IFNG, CCL3 and CCL11). The low frequency of cytokines/chemokines/growth factors detection is evident in T1DM patients. The symbol Ø identifies samples that were not available for the experiment.

11 analytes (IL1B, IL2, IL5, IL6, IL8, IL10, IL13, IL15, CSF2, VEGF and TNF) were undetectable in all samples tested. 10 analytes (IL9, IL12, IL17, FGF, IFNG, CCL3, CCL11, ILR1A, IL4 and PDGF) were sporadically detected in control and T1DM patients; cytokines tended to be less frequently detected in T1DM patients (Fig. 8). Due to the variable nature of the data, statistical comparison is not straightforward. Cytokine analysis was done on unstimulated samples, which may explain low/undetectable values. With this consideration, in T1DM patients, IFNG was less frequently detected in the 1^st^, 2^nd^ and 3^rd^ trimesters compared to control patients. Although non-conclusive, six analytes (IL7, CSF3, CXCL10, CCL2, CCL4 and CCL5) were detected in all samples and statistically analyzed. Only plasma IL7 and CSF3 were significantly lower in T1DM than control patients at 2^nd^ trimester (Table 2). IL7 supports the differentiation and functions of multiple lymphoid and myeloid lineages [49,50]; including CD56^bright^ NK cells [51], but knowledge concerning the role of IL7 during pregnancy is limited. However, a potential role for IL7 in induction of expression of CXCR3, CXCR4 and other integrins is reported in T lymphocytes [52–54]. This may be linked with the increased number of homing receptors expressed by blood CD56^bright^ NK cells in control, but not in T1DM patients. Larger number of patients and *in vitro* studies would be required to address this possible mechanism. CSF3 was also up regulated in control patients at 2^nd^ trimester, but not in T1DM (Table 2). Although more frequently studied during labor [55], CSF3 is reported to promote Type 2 cytokine secretion, activate T regulatory cells [56], and stimulate endometrial vascular remodeling *ex-vivo* [57]. These functions could aid in coordination of the shift to Type 2 immune state observed in control patients in 2^nd^ trimester, which is not seen in T1DM patients. Confirmatory and functional studies are necessary to establish the importance and mechanistic roles for CSF3 deficiency in T1DM pregnancies.

**Table 2:** Cytokine concentrations in control and T1DM plasma across pregnancy and postpartum.

Cytokine	1^st^ trimester					2^nd^ trimester					3^rd^ trimester					Postpartum				
	Control		T1DM			Control		T1DM			Control		T1DM			Control		T1DM		
	Median (pg|ml)	IQR	Median (pg|ml)	IQR	*P*	Median (pg|ml)	IQR	Median (pg|ml)	IQR	*P*	Median (pg|ml)	IQR	Median (pg|ml)	IQR	*P*	Median (pg|ml)	IQR	Median (pg|ml)	IQR	*P*
IL7	5.1	3.1	1.7	6.05	***0*.*17***	***4*.*6***	***7*.*3***	***1*.*0***	***2*.*3***	***0*.*03***	3.9	5.2	0.7	2.2	***0*.*10***	6.1	15.7	5.4	7.5	***0*.*42***
CSF3	67.2	41.0	17.2	85.8	***0*.*11***	***81*.*1***	***100*.*7***	***21*.*5***	***32*.*3***	***0*.*04***	60.1	63.3	17.2	45.2	***0*.*14***	41.5	152.9	104.6	131.8	***0*.*69***
CXCL10	367.0	147.2	381.4	421.8	***0*.*79***	350.5	116.7	358.1	210.1	***0*.*24***	413.1	68.4	339.3	230.5	***0*.*06***	362.5	122.8	318.5	426.2	***0*.*69***
CCL2	21.2	10.7	26.3	7.0	***0*.*66***	25.9	17.4	21.4	9.2	***0*.*58***	24.3	14.3	15.5	6.7	***0*.*06***	31.9	10.9	29.5	11.0	***0*.*69***
CCL4	33.9	19.2	38.2	24.3	***0*.*42***	34.0	22.2	30.9	15.8	***0*.*69***	38.0	20.1	31.9	5.9	***0*.*24***	40.2	23.2	36.9	15.5	***1***
CCL5	384.8	509.1	89.52	238.1	***0*.*08***	324.2	342.7	172.4	346.5	***0*.*58***	306.6	363.3	93.38	295.2	***0*.*39***	448.2	833.2	243.1	916.4	***0*.*91***

Mann-Whitney-U test (non-normal distribution; P < 0.05 found only for IFNG in the 1^st^ trimester. T1DM calculations shown include women who became preeclamptic in 3^rd^ trimester.

## Conclusions

This study demonstrates that the impact of normal pregnancy on circulating NK and T cell subsets is not uniform. In control women, NK and T cell subsets convert from Type 1 to Type 2 immune dominance in 2^nd^ trimester. Amongst these subsets, CD56^bright^ NK cells from normal pregnant women express the highest levels of receptors for extravasation and chemokine-directed movement into tissues and this expression is temporally dynamic. In T1DM gestations, no conversion to Type 2 immune profile was observed amongst the eight NK and T cell subsets examined. Thus, a pro-inflammatory systemic environment is maintained throughout T1DM pregnancies, although plasma IFNG, IL7 and CSF3 were lower in TIDM than control patients. Further, in T1DM women, reduced expression of key receptors for endothelial cell interactions and decidual homing occurs during 2^nd^ trimester in circulating Type 2 CD56^bright^ NK cells. These alterations may reduce normal lymphocyte homing into decidua. A sustained Type 1 pro-inflammatory milieu in the periphery is a known deviation from healthy gestation. Understanding the mechanisms causing the heightened Type 1 status of pregnant T1DM women would likely reveal potential therapeutic targets. The goal would be to promote Type 2 CD56^bright^ NK cell homing to decidua where pro-angiogenic, anti-inflammatory functions are thought to promote physiological gestations, free from complications [58].

## Supporting Information

S1 Table Panel of antibodies used for flow cytometric analyses of lymphocyte subsets. All antibodies were monoclonal mouse anti-human.
(DOC)

S2 Table Flow cytometry-staining strategy. A total of 18 tubes were read for each patient. Compensation was completed post-acquisition individually for each multiple color sample.
(DOC)

S3 Table Type 1 lymphocyte (IL18R1+) and Type 2 lymphocyte (IL1RL1+) percentage in control and T1DM patients across pregnancy and postpartum. Mean±SD of percentage of Type 1 and Type 2 lymphocyte subsets. * Significance between periods (1st, 2nd, 3rd trimester and postpartum within each patient group); # significance between patients (control and T1DM within a specific test interval). P<0.05.
(DOC)

S4 Table Adhesion molecule and chemokine receptor expression by Type 1 blood lymphocytes across pregnancy and postpartum. Data are percentage mean ± SEM of Type 1 cells expressing a specific adhesion molecule or chemokine receptor. * P<0.05 and ** P<0.01. Significance between periods (1st, 2nd, 3rd trimester and postpartum within each patient group) is not shown in this table. T1DM calculations shown include women who became preeclamptic in 3rd trimester.
(DOC)

S5 Table Adhesion molecule and chemokine receptor expression by Type 2 blood lymphocytes across pregnancy and postpartum. Data are percentage mean ± SEM of Type 2 cells expressing a specific adhesion molecule or chemokine receptor. * P<0.05 and ** P<0.01. Significance between periods (1st, 2nd, 3rd trimester and postpartum within each patient group) is not shown in this table. T1DM calculations shown include women who became preeclamptic in 3rd trimester.
(DOC)
