# Supplementary material for: Homing Receptor Expression Is Deviated on CD56+ Blood Lymphocytes during Pregnancy in Type 1 Diabetic Women
Source: PLoS One. 2015 Mar 20;10(3):e0119526. doi: 10.1371/journal.pone.0119526 (PMC4368780; doi:10.1371/journal.pone.0119526)
Supplement: S1 Table — All antibodies were monoclonal mouse anti-human. (DOC) [file pone.0119526.s001.doc]

| **Table S1. Panel of antibodies used for flow cytometric analyses of lymphocyte subsets.** | | | |
| --- | --- | --- | --- |
| **Antibody** | **Dilution** | **Source** | **Target** |
| Anti-CD3-PE-Cy7 | 1:50 | Beckman Coulter, Mississauga, ON, Canada | T and NKT cells |
| Anti-CD56-PE-Cy5 | 1:10 | Immunotech, Beckman Coulter, Mississauga, ON, Canada | NK and NKT cells |
| Anti-IL18R-FITC | 1:5 | eBioscience, San Diego, CA, USA | Pan type 1 cytokine marker |
| Anti-IL1RL1-FITC | undiluted | MDBiosciences, St. Paul, MN, USA | Pan type 2 cytokine marker |
| Anti-CXCR3-PE | undiluted | R&D Systems, Inc., Minneapolis, MN, USA | Chemokine receptor |
| Anti-CXCR4-PE | undiluted | R&D Systems, Inc., Minneapolis, MN, USA | Chemokine receptor |
| Anti-ITGA4-PE | undiluted | Biolegend, San Diego, CA, USA | α4 integrin adhesion molecule |
| Anti-SELL-PE | 1:5 | Biolegend, San Diego, CA, USA | L-selectin homing molecule |
| Mouse IgG1-PE-Cy7 | 1:50 | Biolegend, San Diego, CA, USA | Isotype control |
| Mouse IgG1-PE-Cy5 | 1:10 | Biolegend, San Diego, CA, USA | Isotype control |
| Mouse IgG1-FITC | 1:5 | Biolegend, San Diego, CA, USA | Isotype control |
| Mouse IgG1-PE | undiluted | Immunotech, Beckman Coulter, Mississauga, ON, Canada | Isotype control |
| Mouse IgG2-PE | undiluted | R&D Systems, Inc., Minneapolis, MN, USA | Isotype control |

All antibodies were monoclonal mouse anti-human.
