# Supplementary material for: Homing Receptor Expression Is Deviated on CD56+ Blood Lymphocytes during Pregnancy in Type 1 Diabetic Women
Source: PLoS One. 2015 Mar 20;10(3):e0119526. doi: 10.1371/journal.pone.0119526 (PMC4368780; doi:10.1371/journal.pone.0119526)
Supplement: S2 Table — A total of 18 tubes were read for each patient. Compensation was completed post-acquisition individually for each multiple color sample. (DOC) [file pone.0119526.s002.doc]

| **Table S2. Flow cytometry-staining strategy. A total of 18 tubes were read for each patient. Compensation was completed post-acquisition individually for each multiple color sample.** | | | | |
| --- | --- | --- | --- | --- |
| **Fluorochromes** | **PE-Cy5** | **PE-Cy7** | **FITC** | **PE** |
| Unstained | - | - | - | - |
| Isotype | Mouse IgG1 | Mouse IgG1 | Mouse IgG1 | Mouse IgG1, IgG2 |
| CD3 | - | CD3 | - | - |
| CD56 | CD56 | - | - | - |
| IL8R1 | - | - | IL8R1 | - |
| IL1RL1 | - | - | IL1RL1 | - |
| CXCR3 | - | - | - | CXCR3 |
| ITGA4 | - | - | - | ITGA4 |
| SELL | - | - | - | SELL |
| CXCR4 | - | - | - | CXCR4 |
| Four color 1 | CD56 | CD3 | IL8R1 | CXCR3 |
| Four color 2 | CD56 | CD3 | IL1RL1 | CXCR3 |
| Four color 3 | CD56 | CD3 | IL8R1 | ITGA4 |
| Four color 4 | CD56 | CD3 | IL1RL1 | ITGA4 |
| Four color 5 | CD56 | CD3 | IL8R1 | SELL |
| Four color 6 | CD56 | CD3 | IL1RL1 | SELL |
| Four color 7 | CD56 | CD3 | IL8R1 | CXCR4 |
| Four color 8 | CD56 | CD3 | IL1RL1 | CXCR4 |
