# Supplementary material for: Homing Receptor Expression Is Deviated on CD56+ Blood Lymphocytes during Pregnancy in Type 1 Diabetic Women
Source: PLoS One. 2015 Mar 20;10(3):e0119526. doi: 10.1371/journal.pone.0119526 (PMC4368780; doi:10.1371/journal.pone.0119526)
Supplement: S3 Table — Mean±SD of percentage of Type 1 and Type 2 lymphocyte subsets. * Significance between periods (1st, 2nd, 3rd trimester and postpartum within each patient group); # significance between patients (control and T1DM within a specific test interval). P<0.05. (DOC) [file pone.0119526.s003.doc]

| **Table S3. Type 1 lymphocyte (IL18R1^+^) and Type 2 lymphocyte (IL1RL1^+^) percentage in control and T1DM patients across pregnancy and postpartum** | | | | | | | | | | | | | | | | | |
| --- | --- | --- | --- | --- | --- | --- | --- | --- | --- | --- | --- | --- | --- | --- | --- | --- | --- |
|  | **1^st^ trimester** | | | | **2^nd^ trimester** | | | | | **3^rd^ trimester** | | | | **Postpartum** | | | |
|  | **Control** | **T1DM** | | **Control** | | | **T1DM** | | **Control** | | | **T1DM** | | | **Control** | | **T1DM** |
| **Type 1 lymphocyte (IL18R1^+^)** | | | | | | | | | | | | | | | | | |
| **CD56^bright^** | 92.0±4.5 | | 92.8±3.4 | | | 95.0±3.0 | | 93.2±3.7 | | | 86.1±9.1 | | 91.9±6.5 | | 88.3±9.0 | 82.5±9.8 | |
| **CD56^dim^** | 21.9±6.0 | | 20.6±9.3 | | | 28.5±11.9 | | 23.9±7.0 | | | 18.0±9.5 | | 21.6±6.1 | | 18.6±9.8 | 19.4±8.9 | |
| **NKT** | 63.1±21.8 | | 45.6±29.0 | | | 64.7±21.1 | | 51.6±24.3 | | | 63.3±15.2 | | 58.0±21.0 | | 57.8±18.8 | 21.2±9.5 | |
| **T cell** | 8.2±4.0 | | 8.1±5.6 | | | 9.6±6.2 | | 10.5±5.61 | | | 6.3±3.4 | | 9.5±5.3 | | 8.0±4.9 | 5.4±1.6 | |
| **Type 2 lymphocyte (IL1RL1^+^)** | | | | | | | | | | | | | | | | | |
| **CD56^bright^** | 0.5±0.3* | | 2.3±4.5 | | | 8.1±7.6* | | 2.5±4.6 | | | 0.7±0.7* | | 1.7±2.3 | | 1.7±0.9# | 12.0±12.4# | |
| **CD56^dim^** | 1.8±0.9* | | 3.1±2.4 | | | 6.5±4.9* | | 4.1±2.6 | | | 5.3±3.7 | | 6.6±5.8 | | 4.7±2.3 | 5.9±7.1 | |
| **NKT** | 3.4±2.2 | | 1.5±0.6 | | | 10.4±7.8 | | 3.9±1.9 | | | 4.2±1.9 | | 6.5±5.4 | | 7.4±5.6 | 7.3±9.9 | |
| **T cell** | 1.7±1.0* | | 2.5±1.8 | | | 8.8±7.0* | | 6.1±3.5 | | | 2.2±1.3 | | 7.0±7.5 | | 4.2±4.2 | 2.9±2.3 | |
| Mean±SD of percentage of Type 1 and Type 2 lymphocyte subsets. * Significance between periods (1^st^, 2^nd^, 3^rd^ trimester and postpartum within each patient group); # significance between patients (control and T1DM within a specific test interval). P<0.05. | | | | | | | | | | | | | | | | | |
