# Supplementary material for: Homing Receptor Expression Is Deviated on CD56+ Blood Lymphocytes during Pregnancy in Type 1 Diabetic Women
Source: PLoS One. 2015 Mar 20;10(3):e0119526. doi: 10.1371/journal.pone.0119526 (PMC4368780; doi:10.1371/journal.pone.0119526)
Supplement: S5 Table — Data are percentage mean ± SEM of Type 2 cells expressing a specific adhesion molecule or chemokine receptor. * P<0.05 and ** P<0.01. Significance between periods (1st, 2nd, 3rd trimester and postpartum within each patient group) is not shown in this table. T1DM calculations shown include women who became preeclamptic in 3rd trimester. (DOC) [file pone.0119526.s005.doc]

| **Table S5: Adhesion molecule and chemokine receptor expression by Type 2 blood lymphocytes across pregnancy and postpartum.** | | | | | | | | |
| --- | --- | --- | --- | --- | --- | --- | --- | --- |
|  | **Type 2** | | | | | | | |
|  | **1^st^ trimester** | | **2^nd^ trimester** | | **3^rd^ trimester** | | **Postpartum** | |
|  | **Control** | **T1DM** | **Control** | **T1DM** | **Control** | **T1DM** | **Control** | **T1DM** |
| **CD56^bright^** | | | | | | | | |
| **ITGA4** | 0.3±0.3 | 0.8±1.3 | 3.5±3.3 | 2.3±3.5 | 0.1±0.2 | 1.4±2.1 | 0.6±0.4 | 0.8±1.2 |
| **SELL** | 0.3±0.3 | 0.7±1.1 | ***4.7±6.0**** | ***0.9±1.4**** | 0.9±1.6 | 0.6±0.7 | 2.2±2.4 | 1.2±1.1 |
| **CXCR3** | 0.3±0.2 | 1.0±1.7 | ***3.8±2.8***** | ***0.2±0.5***** | 1.2±1.1 | 0.6±0.9 | 0.5±0.6 | 2.6±3.1 |
| **CXCR4** | 0.5±0.7 | 0.8±1.4 | ***5.4±4.8***** | ***0.6±0.8***** | 0.7±1.1 | 1.3±1.2 | 1.5±1.5 | 0.9±1.1 |
| **CD56^dim^** | | | | | | | | |
| **ITGA4** | 1.7±0.9 | 2.3±1.5 | 2.7±1.7 | 3.4±2.4 | 3.5±3.4 | 5.9±6.3 | 4.8±3.1 | 2.1±2.1 |
| **SELL** | 0.9±0.7 | 1.4±1.3 | 2.7±2.2 | 2.0±1.7 | 3.4±1.8 | 3.1±2.4 | 2.9±1.7 | 2.3±2.2 |
| **CXCR3** | 1.0±0.7 | 1.2±1.3 | 1.2±0.5 | 2.0±1.3 | ***3.8±2.3***** | ***0.8±0.4***** | 1.8±1.4 | 1.2±1.2 |
| **CXCR4** | 0.8±0.4 | 1.5±0.9 | 1.8±1.6 | 1.6±1.5 | 3.6±2.7 | 2.3±1.9 | 2.5±1.3 | 1.3±0.2 |
| **NKT** | | | | | | | | |
| **ITGA4** | 2.7±2.1 | 1.7±0.6 | 5.3±3.6 | 4.4±3.5 | 2.5±2.2 | 6.0±5.9 | 3.8±1.9 | 5.1±4.6 |
| **SELL** | 1.4±0.8 | 1.8±2.7 | 4.4±3.3 | 1.5±0.8 | 2.4±2.4 | 3.2±2.7 | 2.9±1.4 | 4.0±5.2 |
| **CXCR3** | 2.7±2.9 | 1.3±0.7 | 5.1±3.4 | 2.9±2.3 | 3.2±1.1 | 2.5±2.6 | 2.8±2.2 | 0.8±0.6 |
| **CXCR4** | 1.3±1.2 | 1.2±1.4 | 2.6±1.3 | 1.6±1.7 | 2.4±1.4 | 3.5±3.6 | 1.3±0.6 | 0.4±0.3 |
| **T cell** | | | | | | | | |
| **ITGA4** | 1.7±1.7 | 1.8±1.0 | 4.1±3.0 | 5.2±3.8 | 1.2±0.7 | 4.1±5.2 | 2.5±2.3 | 3.5±4.0 |
| **SELL** | 1.8±1.6 | 1.8±1.4 | 6.2±6.2 | 5.0±6.0 | 2.1±2.2 | 6.0±6.5 | 4.5±3.6 | 3.3±2.4 |
| **CXCR3** | 1.6±1.7 | 1.8±1.5 | 3.6±3.4 | 3.7±2.6 | 1.2±0.2 | 1.7±1.8 | 1.6±1.3 | 1.4±1.2 |
| **CXCR4** | 1.4±2.2 | 0.6±0.6 | 3.7±3.6 | 1.5±1.1 | 0.7±0.6 | 2.9±2.7 | 2.3±2.6 | 1.2±0.7 |
| Data are percentage mean **±** SEM of Type 2 cells expressing a specific adhesion molecule or chemokine receptor. * **P<0.05 and ** P<0.01.** Significance between periods (1^st^, 2^nd^, 3^rd^ trimester and postpartum within each patient group) is not shown in this table. T1DM calculations shown include women who became preeclamptic in 3^rd^ trimester. | | | | | | | | |
